# Effects of Temperature on Bacterial Communities and Metabolites during Fermentation of Myeolchi-Aekjeot, a Traditional Korean Fermented Anchovy Sauce

**DOI:** 10.1371/journal.pone.0151351

**Published:** 2016-03-15

**Authors:** Ji Young Jung, Hyo Jung Lee, Byung Hee Chun, Che Ok Jeon

**Affiliations:** Department of Life Science, Chung-Ang University, Seoul, 06974, Republic of Korea; Agricultural University of Athens, GREECE

## Abstract

Myeolchi-aekjeot (MA) in Korea is produced outdoors without temperature controls, which is a major obstacle to produce commercial MA products with uniform quality. To investigate the effects of temperature on MA fermentation, pH, bacterial abundance and community, and metabolites were monitored during fermentation at 15°C, 20°C, 25°C, and 30°C. Initial pH values were approximately 6.0, and pH values increased after approximately 42 days, with faster increases at higher temperatures. Bacterial abundances increased rapidly in all MA samples after quick initial decreases during early fermentation and then they again steadily decreased after reaching their maxima, which were significantly greater at higher temperatures. Bacterial community analysis revealed that *Proteobacteria* and *Tenericutes* were predominant in all initial MA samples, but they were rapidly displaced by *Firmicutes* as fermentation progressed. *Photobacterium* and *Mycoplasma* belonging to *Proteobacteria* and *Tenericutes*, respectively, which may include potentially pathogenic strains, were dominant in initial MA, but decreased with the growth of *Chromohalobacter*, which occurred faster at higher temperatures––they were dominant until 273 and 100 days at 15°C and 20°C, respectively, but not detected after 30 days at 25°C and 30°C. *Chromohalobacter* also decreased with the appearance of subsequent genera belonging to *Firmicutes* in all MA samples. *Tetragenococcus*, halophilic lactic acid bacteria, appeared predominantly at 20°C, 25°C, and 30°C; they were most abundant at 30°C, but not detected at 15°C. *Alkalibacillus* and *Lentibacillus* appeared as dominant genera with the decrease of *Tetragenococcus* at 25°C and 30°C, but only *Lentibacillus* was dominant at 15°C and 20°C. Metabolite analysis showed that amino acids related to tastes were major metabolites and their concentrations were relatively higher at high temperatures. This study suggests that high temperatures (approximately 30°C) may be appropriate in MA fermentation, in the light of faster disappearance of potentially pathogenic genera, higher amino acids, growth of *Tetragenococcus*, and faster fermentation.

## Introduction

Fish sauces are amber-colored liquid condiments with a salty taste and distinctive fish flavor produced by the spontaneous fermentation of salted whole small fish (e.g., anchovy and sand eel) [1, 2]. The spontaneous fermentation of fish sauces without the use of starter cultures leads to the growth of various microorganisms, which are primarily derived from raw materials (fish and solar salts) used for the preparation of fish sauces. To date, various halophilic or halotolerant bacteria, such as *Lentibacillus*, *Alkalibacillus*, *Salinimicrobium*, *Halomonas*, *Brevibacterium*, *Halobacterium*, *Staphylococcus*, *Halanaerobium*, and *Tetragenococcus* species, have been identified from fish sauces through culture-dependent and -independent approaches [3–7].

Myeolchi-aekjeot (MA) is a representative traditional Korean fermented fish sauce, which is usually produced by long-term fermentation (more than 6 months) of highly salted anchovies [approximately 25% (w/v)] outdoors without temperature controls. Because fermentation temperature is an important factor that influences microbial growth and enzyme activity during fish sauce fermentation [8, 9], lack of temperature control during the MA fermentation process may be a major cause of inconsistent quality in MA products. However, to the best of our knowledge, intensive scientific studies on the effects of temperature on MA fermentation have not yet been reported.

The main objective of this study was to investigate the effects of temperature on MA fermentation by comparing the bacterial communities and metabolites of MA samples during fermentation at different temperatures (15°C, 20°C, 25°C, and 30°C). Because it has been suggested that microorganisms as well as diverse endogenous enzymes, such as proteinases and lipases, derived from fish raw materials are responsible for the development of tastes and flavors of fish sauces during fermentation [8, 10–12], the metabolites of fermented seafood may reflect a collective phenotypic view of microbial flora and endogenous enzymes. Therefore, a parallel investigation of microbial communities and metabolites during the fermentation process of seafood is indispensable to understanding how microbial flora and endogenous enzymes are involved in seafood fermentation [7, 9, 13–17].

Pyrosequencing of the 16S rRNA gene is a powerful approach to unravel complex microbial communities, including unculturable microbes, and proton nuclear magnetic resonance (^1^H-NMR) is one of the most comprehensive and nondestructive methods for the simultaneous analysis of multiple compounds, especially in fermented foods [9, 15, 17–21]. Because fermentation of salted seafood usually occurs under high-salt conditions with approximately 25% (w/w) salts, *Archaea* may play an important role in salted seafood fermentation [22]; recently, however, the contribution of *Archaea* has been disputed [9, 15, 17]. Therefore, in this study, we investigated the effects of temperature on MA fermentation by pyrosequencing and ^1^H-NMR analyses of the bacterial community and metabolite changes during fermentation.

## Materials and Methods

### Ethic Statement

A field test was not performed in this study. Salted anchovy samples were prepared in the laboratory using anchovies that was bought from a market in Wando of Korea. No specific permissions were required for this study because anchovies were not protected or endangered species.

### Preparation of myeolchi-aekjeot and sampling

Four sets of triplicated myeolchi-aekjeot (MA) samples for four different temperature conditions (total 12 MA samples) were prepared according to a traditional manufacturing method described previously, to be approximately 25% (w/v) equilibrated NaCl concentration [7]. In brief, fresh whole anchovies (called myeolchi in Korean, *Engraulis japonicus*), approximately 5–8 cm in length, that were caught at once from a sea point of the South Sea near Wando, South Korea, were equally dispensed into 12 plastic containers, each containing 1.0 kg anchovies and 270 g solar salts (Shinan, Korea). An additional 400 ml of 25% (w/v) solar salt solution was poured into each container. The prepared 12 MA samples were incubated at 15°C, 20°C, 25°C, and 30°C, with three containers for each temperature. Four milliliters of MA soups (the liquid parts of the MA samples) were intermittently sampled from each container, and their pH values were immediately measured. The MA soups were filtered through four layers of sterile coarse gauze (Daehan, Korea) to remove large particles, and microorganisms were harvested by centrifugation (8000 rpm for 20 min at 4°C) of the filtrates. Microorganisms harvested from three containers at the same temperature were combined and stored at –80°C for bacterial community analysis, and the supernatants were stored separately at –80°C for metabolite analyses. For the measurements of bacterial abundance using quantitative real-time PCR (qPCR) in MA samples, two milliliters of MA soups were additionally sampled and microorganism pellets were harvested by centrifugation as described above. However, the microorganism pellets from each sample were stored separately at –80°C without combining microorganism pellets from the triplicated MA samples unlike the bacterial community analysis. NaCl concentrations in MA samples were measured using the Mohr method [23].

### Quantitative real-time PCR

Bacterial abundances in MA samples were quantitatively estimated using qPCR based on the 16S rRNA gene copies according to the previously described procedure [17]. Briefly, 100 ng of salmon testes DNA (Sigma, USA) was added into the microorganism pellets as an exogenous and internal standard. Total genomic DNA was extracted using a FastDNA Spin kit (MPbio, USA) according to the manufacturer’s instructions and qPCR was performed using the bacterial 16S rRNA gene-targeting primer set, bac340F (5'- CCT ACG GGA GGC AGC AG-3')/bac758R (5’-CTA CCA GGG TAT CTA ATC C-3'). Sample-to-sample variations caused by different DNA extraction and PCR amplification efficiencies were normalized based on qPCR using the primer set, Sketa2-F (5'-GGT TTC CGC AGC TGG G-3')/Sketa2-R (5'-CCG AGC CGT CCT GGT CTA-3'), targeting the rRNA gene operon of added salmon testes DNA. A standard curve for the calculations of bacterial 16S rRNA gene copies was generated from pCR2.1 vectors (Invitrogen, USA) carrying bacterial (*Salimicrobium*) 16S rRNA gene derived from a fermented fish [15]. All of the qPCR assays were performed in triplicate as described previously [24].

### Pyrosequencing, sequence processing, and data analysis

Genomic DNA extraction from MA samples, barcoded pyrosequencing of bacterial 16S rRNA genes, and processing of pyrosequencing reads were performed according to previously described procedures [17]. Briefly, total genomic DNA was extracted from the combined pellets of the triplicate samples using the Fast-DNA Spin kit (MPbio). V1 to V3 regions of the bacterial 16S rRNA genes were amplified using the primer set, Bac27F (5'-adaptor B-AC-GAGTTT GAT CMT GGC TCA G-3')/Bac541R (5'-adaptor A-X-AC-WTT ACC GCG GCT GCT GG-3'), where X denotes unique 7–11 barcode sequences (S1 Table), as described previously [25]. PCR products were purified using a PCR purification kit (Bioneer, Korea) and quantified using a SynergyMx ELISA reader equipped with a Take3 multivolume plate (BioTek, USA). Composite samples for pyrosequencing were prepared by pooling equal amounts of the purified PCR products and their pyrosequencing was conducted using a 454 GS-FLX Titanium system (Roche, Germany) at Macrogen (Korea).

Pyrosequencing reads obtained were processed using the RDP pyrosequencing pipeline (http://pyro.cme.msu.edu/) [26]. The sorting of pyrosequencing reads according to MA samples was conducted using their barcode sequences, and then the barcode sequences were removed. Pyrosequencing reads with more than two ‘N’ (unknown nucleotides), shorter than 300 bases, or below 20 (error rate 0.01) average quality values were excluded from further analysis. Putative chimeric reads were removed using the chimera.slayer command of the MOTHUR program [27]. From the resulting high-quality pyrosequencing reads, a calculation of operational taxonomic units (OTU), Shannon-Weaver and Chao1 richness indices, and evenness, and rarefaction analysis were conducted using the RDP pyrosequencing pipeline (http://pyro.cme.msu.edu/) [26]. To compare changes in the bacterial communities of MA samples incubated at different temperatures throughout the entire fermentation period, a principal-component analysis (PCA) was carried out using PLS_Toolbox v4.0 (Eigenvector Research Inc., USA) in MATLAB version R2009a software (The MathWorks Inc., USA). Briefly, all high-quality sequencing reads derived from MA samples were merged and clustered into respective OTUs by using the RDP pyrosequencing pipeline at a 97% sequence similarity. OTU information containing read numbers for respective MA samples was imported into the MATLAB program and a score plot was constructed using mean-centered scaling in the PLS_Toolbox v4.0. The PCA was performed separately using the sequence data sets both before and after removing singletons as described by Zhou et al. [28].

The taxonomic classifications of the high-quality sequencing reads derived from MA samples incubated at different temperatures were performed using the RDP naïve Bayesian rRNA Classifier 2.6 [29] at the phylum and genus levels with an 80% confidence threshold.

### Metabolite analysis using ^1^H-NMR and data analysis

Metabolites in MA samples were analyzed using a ^1^H-NMR approach according to a previously described procedure [25]. Briefly, MA supernatants that were stored at –80°C for metabolite analyses were thawed and 10-fold diluted with sterile distilled water, and adjusted to a pH value of 6.0. One milliliter of the diluted supernatants was lyophilized and dissolved in 600 μl of deuterium oxide (Sigma, USA) with 5 mM sodium 2,2-dimethyl-2-silapentane-5-sulfonate (Sigma, USA). The resulting solutions were transferred into 5-mm NMR tubes, and their ^1^H NMR spectra were acquired using a Varian Inova 600-MHz NMR spectrometer (Varian, USA). To compare changes in all metabolites of MA samples incubated at different temperatures, a PCA was performed using the PLS_Toolbox v4.0. Briefly, all NMR spectra from MA samples were phased, baseline-corrected, and water signal-suppressed using MestReNova v8.1 software (Mestrelab Research SL, Spain). The NMR spectral data of 0.5 to 10.0 ppm were binned into 0.001-ppm spectral buckets and normalized to the total spectral area. The NMR spectra were converted to a script format and the mean values of the NMR spectra derived from the triplicate analyses were calculated. Finally, a PCA score plot was constructed using mean-centered scaling in PLS_Toolbox v4.0.

Identification and quantification of metabolites from the ^1^H-NMR spectra of MA samples were performed using the Chenomx NMR Suite (v6.1; Chenomx, Canada) based on peak areas of 2,2-dimethyl-2-silapentane-5-sulfonate as the internal standard.

### Sequencing data accession number

The sequence data of the 16S rRNA genes from this study are publicly available in the NCBI Short Read Archive under accession no. SRP064726 (NCBI BioProject PRJNA298509).

## Results

### General features of MA fermentation

**Fig 1:**
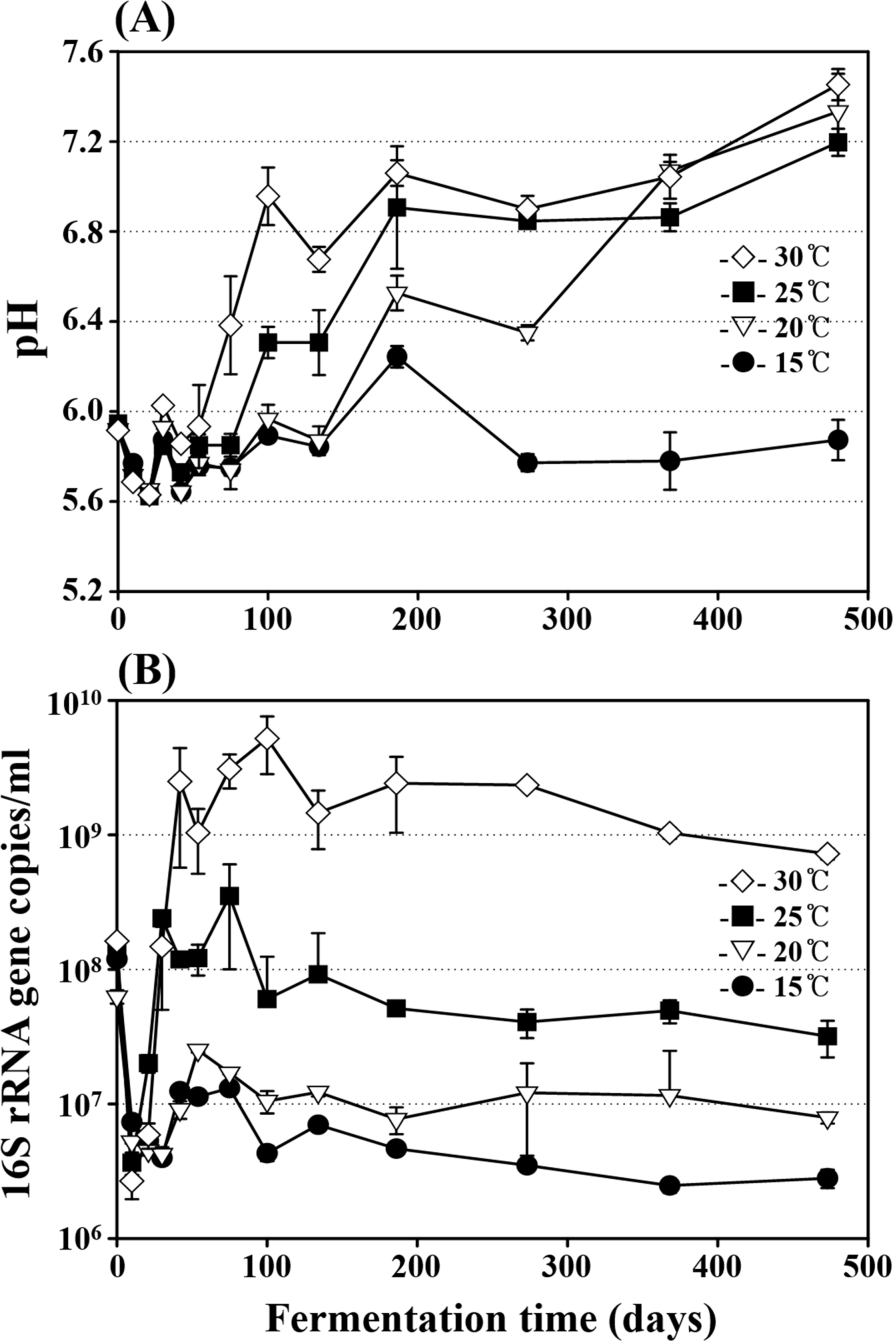
**Profiles of pH (A) and bacterial 16S rRNA gene copies (B) during the entire fermentation period in MA samples incubated at 15°C, 20°C, 25°C, and 30°C.** The measurements were performed in triplicate, and the error bars indicate the standard deviations.

Four sets of MA samples were incubated for 473 days at 15°C, 20°C, 25°C, and 30°C, respectively, and their NaCl concentrations were held nearly constant at approximately 24.7±0.5% (w/v) in all MA samples throughout the entire fermentation period. Initial pH values of the MA samples were approximately 6.0 (Fig 1A). The pH values began to increase after approximately 42 days of fermentation, and the pH increases were faster at higher temperatures. The pH values of the 20°C, 25°C, and 30°C MA samples increased to 7.2–7.4 at the end of fermentation, while those of the 15°C MA samples decreased again to below 5.8 after around 200 days of fermentation.

Although the exact enumeration of viable cells using qPCR is impossible because MA samples contain many dead cells and the numbers of 16S rRNA gene operons vary depending on species or strains [30], qPCR is currently one of most appropriate approaches to estimate bacterial abundance in MA samples containing many unculturable bacteria [7]. Therefore, bacterial abundance in the MA samples was monitored over the entire fermentation period using a qPCR approach based on 16S rRNA gene copies (Fig 1B). The initial 16S rRNA gene copies of total *Bacteria* were approximately 1.0 × 10^8^ copies/ml, and copy numbers decreased rapidly to around 5.0 × 10^6^ copies/ml in samples from all temperature during the early fermentation period (Fig 1B). The 16S rRNA gene copies of the 25°C and 30°C MA samples increased after around 10 days to their highest values of approximately 6.0 × 10^8^ and 7.0 × 10^9^ copies/ml at around 75 and 100 days of fermentations, respectively. Whereas the 16S rRNA gene copies of the 15°C and 20°C MA samples increased after around 30 days to their highest values of approximately 1.0 × 10^7^ and 4.0 × 10^7^ copies/ml at around 54 and 42 days of fermentation, respectively. After the bacterial 16S rRNA gene copies reached their maximum values, they decreased slowly and gradually in samples at all temperatures until the end of MA fermentation.

### Bacterial diversities in the MA samples fermented at different temperatures

**Fig 2:**
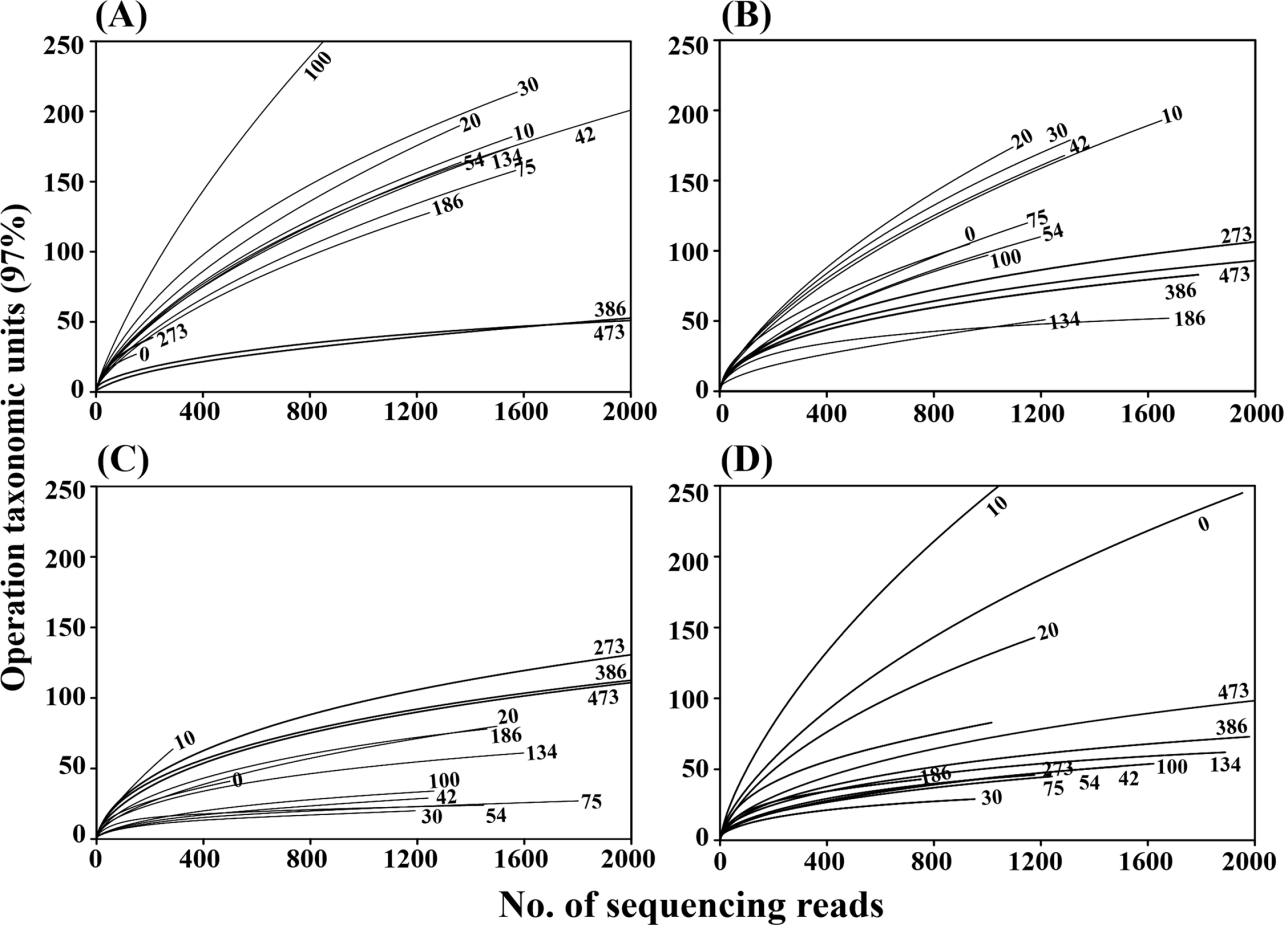
**Rarefaction curves showing the bacterial diversity of MA samples fermented at 15°C (A), 20°C (B), 25°C (C), and 30°C (D).** The rarefaction curves were depicted by the RDP pipeline with a 97% OTU (operational taxonomic units) cutoff using 16S rRNA gene sequences. The x- and y-axes indicate the numbers of sequencing reads sampled and the cumulative numbers of OTU recorded, respectively. Numbers beside the curves represent the fermentation time (days).

A barcoded pyrosequencing approach was applied to analyze bacterial diversity and community structure in the MA samples fermented at 15°C, 20°C, 25°C, and 30°C, and a total of 164,673 16S rRNA gene sequencing reads were generated from the 52 MA samples. After removing barcoded PCR primers and low quality reads including chimera, 98,495 clean reads were used for further bacterial diversity and community analyses (S2 Table). Although the same anchovies were used for the MA sample preparation, bacterial diversities of rarefaction curves at 0 day were a little different, which might be caused by the differences in the genomic DNA used for PCR––the differences of the genomic DNA at 0 day might be caused by incomplete mixing. However, the rarefaction curves clearly showed that bacterial diversity increased during the early MA fermentation period in samples at all temperatures, and then the bacterial diversity decreased as fermentation proceeded. Bacterial diversity decreased more quickly in higher temperature MA samples, and the lowest diversities were reached at 30, 30, 134, and 386 days of fermentations in the 30°C, 25°C, 20°C, and 15°C MA samples, respectively. Bacterial diversities increased again in MA samples at all temperatures during the late fermentation period. The changes in bacterial diversity during MA fermentation occurred more rapidly at higher temperatures than at lower temperatures (Fig 2). The statistical diversity indices, including OTU, Shannon–Weaver, and Chao1, supported the results of the rarefaction analysis, although the number of sequencing reads obtained affected the index values (S2 Table).

### Statistical comparisons of bacterial community and metabolite changes in the MA samples fermented at different temperatures

**Fig 3:**
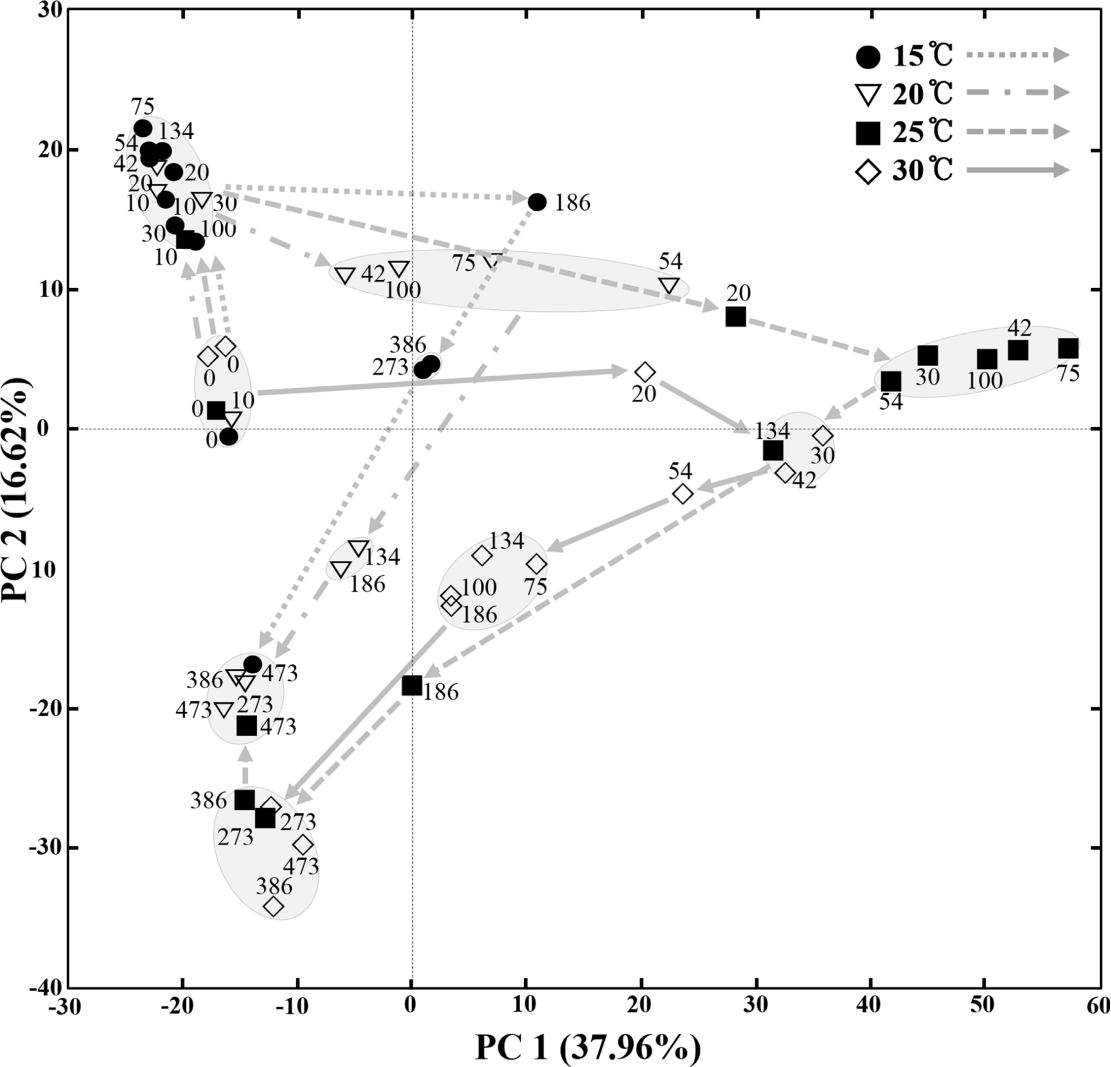
A score plot of principal-component analysis (PCA) showing the bacterial community changes of MA samples incubated at 15°C, 20°C, 25°C, and 30°C during the entire fermentation period. Numbers beside the symbols represent the fermentation time (days) of MA samples. The curved arrows indicate the routes of the bacterial community changes during the fermentation period in MA samples fermented at different temperatures. The score plot was constructed using weighted OTU information in respective MA samples.

A principal-component analysis (PCA) based on all OTUs derived from the MA samples fermented at different temperatures was conducted to statistically compare bacterial community changes over the entire fermentation period (Fig 3). The PCA results showed that changes in the bacterial communities depended on the fermentation temperatures. Bacterial community changes at 15°C, 20°C, and 25°C occurred similarly during the initial fermentation period, but then diverged at later stages of fermentation. The bacterial community changes occurred slowly and slightly at 15°C, while at 30°C, the bacterial community changes occurred more rapidly and in a different pattern from those of other temperatures. The bacterial communities in the 25°C and 30°C samples appeared similar in the middle of fermentation process, but eventually their bacterial communities also diverged. However, the bacterial communities in the 15°C, 20°C, and 30°C samples diverged in the middle of fermentation process, but their eventual bacterial communities appeared similar. Recently, it has been demonstrated that amplicon-based community analyses can suffer from low reproducibility, often due to data distortion caused by noise associated with singleton reads [28]. Therefore, a PCA was also performed using sequence data sets with the removal of singletons; the results showed the similar patterns to those seen in the PCA (S1 Fig).

**Fig 4:**
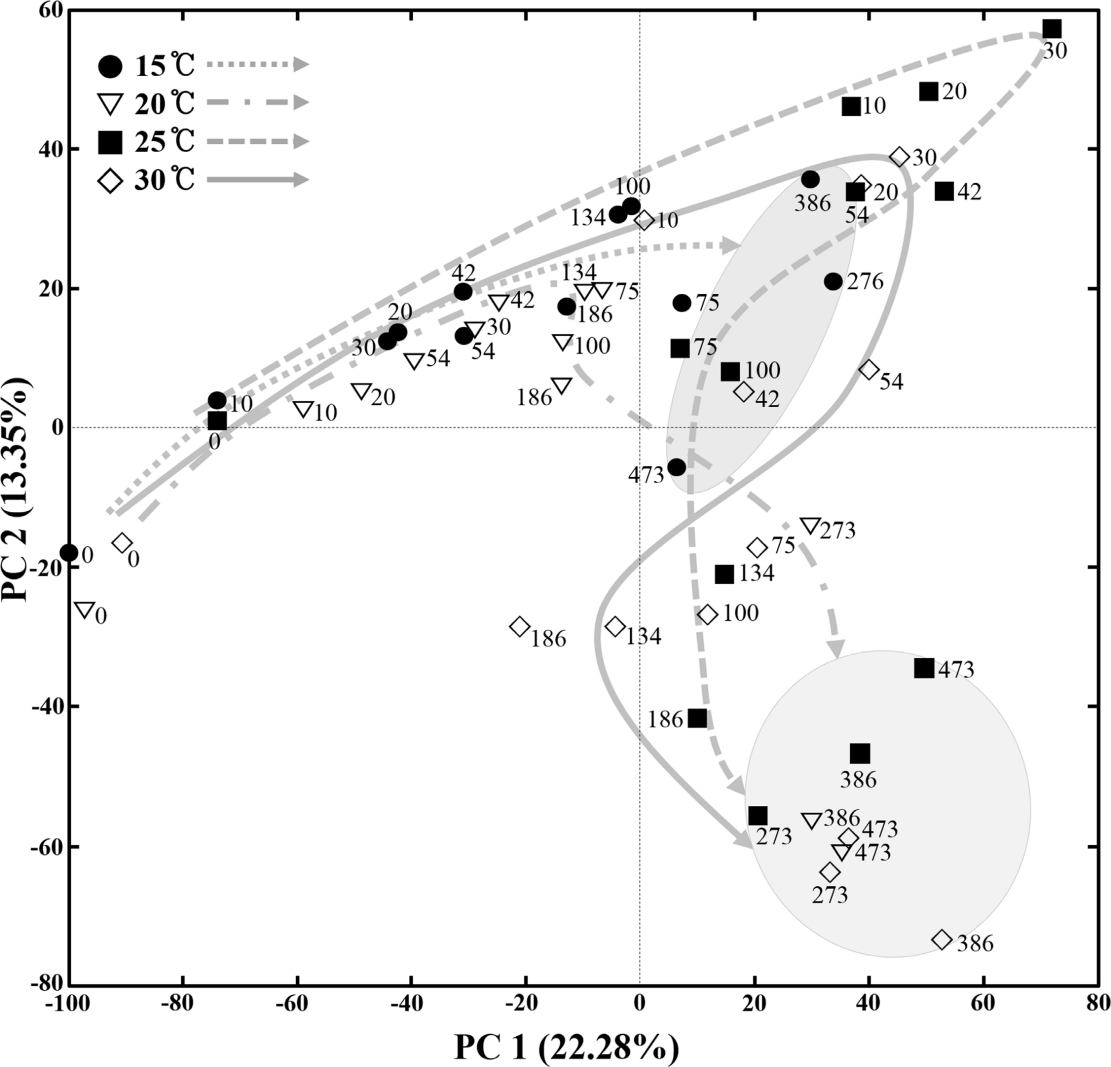
A score plot of principal-component analysis (PCA) showing the metabolite changes of MA samples incubated at 15°C, 20°C, 25°C, and 30°C during the entire fermentation period. Numbers beside the symbols represent the fermentation time (days) of MA samples. The score plot was constructed using the mean values of all ^1^H-NMR peaks obtained from triplicate analyses. The curved arrows indicate the routes of the metabolite changes during the fermentation period in MA samples fermented at different temperatures.

The PCA using NMR peaks representing all metabolites, including carbohydrates, amino acids, organic acids, and other organic compounds, present in the MA samples showed that metabolite changes occurred differently in the 15°C, 20°C, 25°C, and 30°C MA samples (Fig 4). Specifically, the metabolite changes occurred very slowly in the 15°C MA samples and their metabolite compositions were clearly distinguished from those of other temperature samples by the end of the fermentation process. However, in the 20°C, 25°C, and 30°C MA samples, although metabolite changes differed, their eventual metabolite compositions became relatively similar at the end of fermentation. The PCA results suggest that bacterial community changes as well as metabolite changes depend on the temperature during MA fermentation.

### Bacterial communities in the MA samples fermented at different temperatures

**Fig 5:**
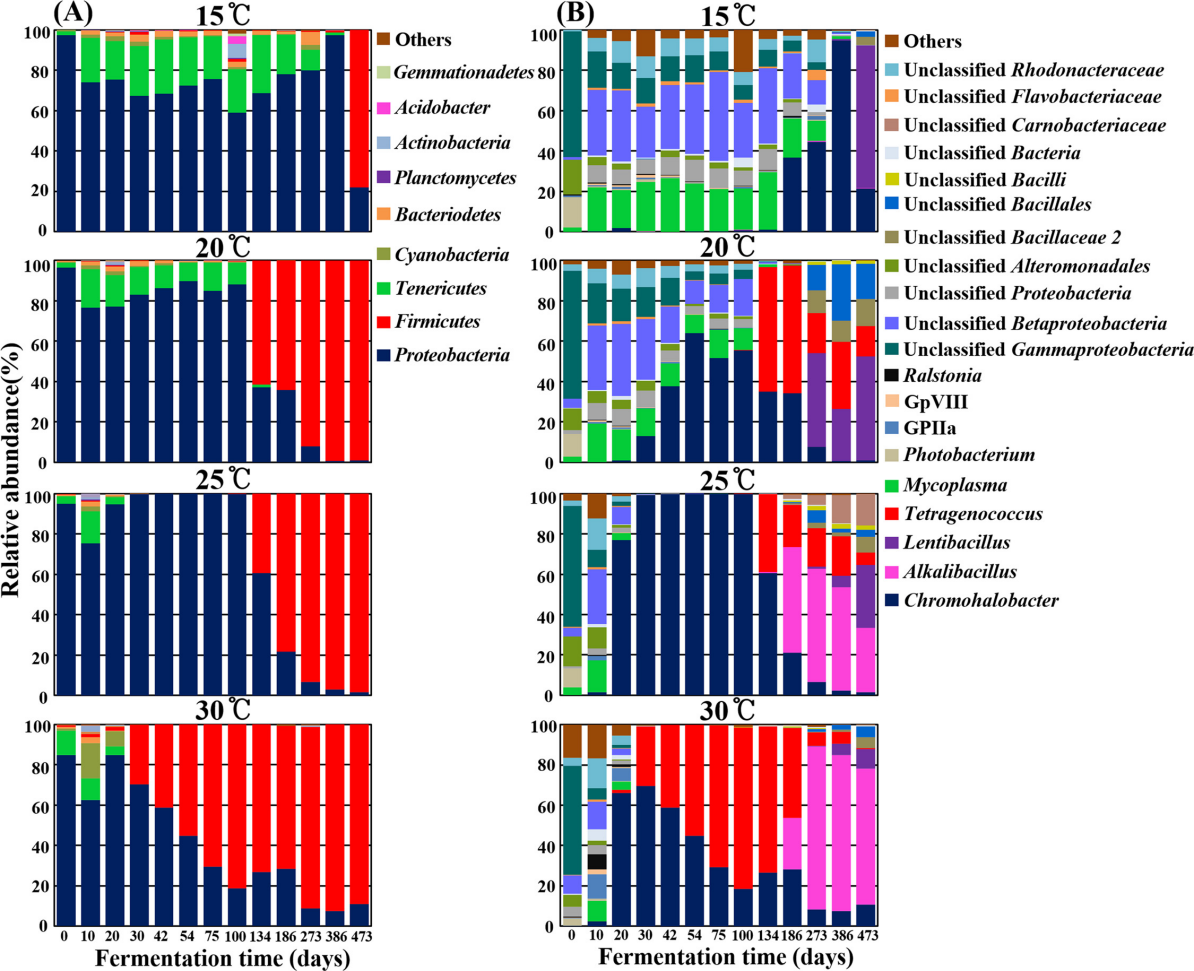
**Bacterial taxonomic compositions showing the bacterial successions of MA samples incubated at 15°C, 20°C, 25°C, and 30°C during the entire fermentation period at the phylum (A) and genus (B) levels.** Others are composed of taxonomic compositions showing less than 1% and 2% of the total reads in respective all samples of the phylum and genus level analyses.

The bacterial pyrosequencing reads were classified at the phylum and genus levels using the RDP classifier to compare changes in communities taxonomically in the MA samples fermented at different temperatures (Fig 5). The phylum level analysis showed that the phyla *Proteobacteria* or *Firmicutes* were predominant in MA samples at all temperatures over the entire fermentation period (Fig 5A). In the initial MA samples (day 0), members of *Proteobacteria* were predominant; those of *Tenericutes* were also detected as a minor group. The phylum *Tenericutes* slightly increased during the early fermentation period, and the increases were faster and higher at lower temperatures. In the 15°C MA samples, the relative abundance of *Tenericutes* increased to 26.8% of total bacteria at 42 days of fermentation, and this dominance was maintained until almost the end of fermentation. In contrast, in the 25°C and 30°C MA samples, *Tenericutes* were identified briefly during early fermentation with a lower relative abundance compared to the lower temperature MA samples. *Tenericutes* were not detected even after 30 days of fermentation in the 25°C and 30°C MA samples. *Proteobacteria* were replaced with *Firmicutes* as the fermentation progressed, and this replacement occurred earlier at higher temperatures. *Proteobacteria* decreased quickly with the increase in *Firmicutes* after 30 days of fermentation in the 30°C MA samples, while they remained predominant until 386 days of fermentation in the 15°C MA samples. The replacement of *Proteobacteria* with *Firmicutes* occurred relatively similarly in the 20°C and 25°C MA samples, but members of *Tenericutes* were detected for a longer period of time in the 20°C MA samples.

The genus level analysis showed that the bacterial community changes depended on fermentation temperatures, as shown in the PCA results of Fig 3 based on all OTUs (Fig 5B). Unclassified *Gammaproteobacteria*, unclassified *Alteromonadales*, and *Photobacterium* that might have been derived primarily from raw anchovies were found to be dominant in the initial MA samples, but unclassified *Betaproteobacteria*, *Mycoplasma*, unclassified *Gammaproteobacteria*, unclassified *Rhodobacteraceae*, and unclassified *Proteobacteria* became dominant at just 10 days of fermentation in all temperature MA samples. After that, the bacterial communities in the MA samples diverged, with community profiles dependent on fermentation temperatures. In the 15°C MA samples, the dominant genera of 10-day MA samples remained dominant until 134 days of fermentation without the appearance of newly dominant genera, while in the 25°C and 30°C MA samples, they became minor populations within only 20 days of fermentation, along with the appearance of *Chromohalobacter*. The genus *Chromohalobacter*, belonging to the phylum *Proteobacteria*, became the most prevalent genus with a decrease in the previously dominant genera in MA samples at all temperatures. As the MA fermentation progressed, other dominant genera appeared with the decrease of *Chromohalobacter*, but the new dominant genera differed depending on fermentation temperatures. The genus *Tetragenococcus*, belonging to the phylum *Firmicutes*, appeared as the major genus in the 20°C, 25°C, and 30°C MA samples. In the 30°C MA samples, *Tetragenococcus* was dominant only within the first 30 days of fermentation, while in the 20°C and 25°C MA samples, *Tetragenococcus* was still detected after 134 days of fermentation. Nevertheless, *Alkalibacillus* appeared as a dominant genus at a similar time (186 days) with a decrease in *Tetragenococcus* in the 25°C and 30°C MA samples, meaning that *Tetragenococcus* was prevalent for a longer period in the 30°C MA samples than in the 25°C MA samples. However, the growth of *Alkalibacillus* was not observed in the 15°C and 20°C MA samples. The genus *Lentibacillus* appeared as the dominant genus in all MA samples, regardless of fermentation temperature, during the late fermentation period, and their relative abundances were higher in the lower temperature MA samples.

### Metabolite profiles in the MA samples fermented at different temperatures

**Fig 6:**
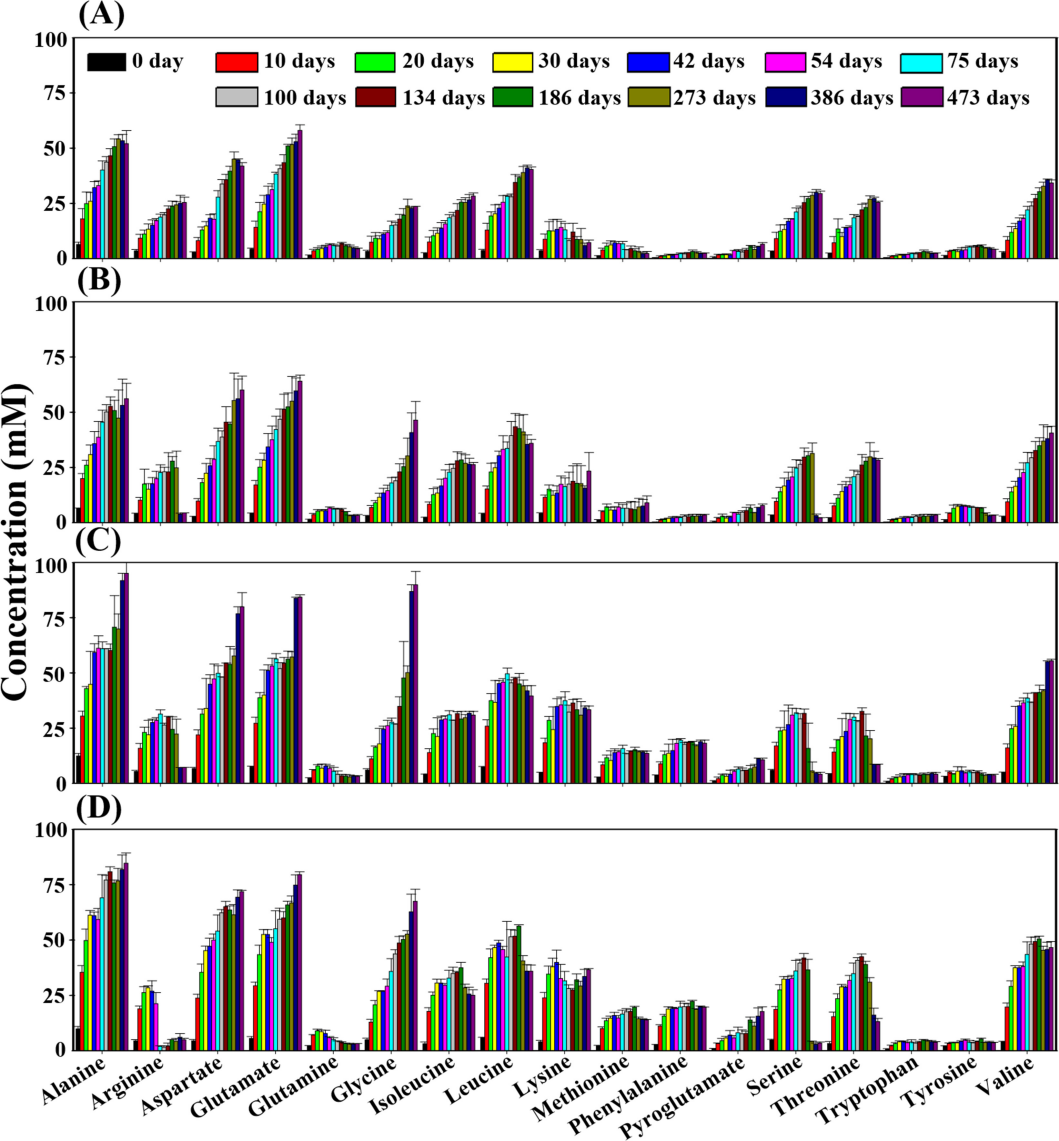
**Changes of amino acids identified from MA fermented at 15°C (A), 20°C (B), 25°C (C), and 30°C (D) during the entire MA fermentation period.** Data are given as the average value ± standard deviation, measured in triplicate. Quantification was determined using the Chenomx NMR Suite v. 6.1 with 2,2-dimethyl-2-silapentane-5-sulfonate as the internal standard.

Metabolite identification and quantification of ^1^H-NMR spectra derived from the MA samples fermented at different temperatures were conducted to investigate changes in the major metabolites, including amino acids, nitrogen compounds, organic acids, and methylamines, over the entire fermentation period. The proteolysis of proteins to amino acids is an important process that enhances flavors and tastes of fish sauces during fermentation. This is accomplished by both endogenous and exogenous proteases, originating from raw materials and halophilic bacteria, respectively [8, 15, 31]. This study showed that amino acids were the major metabolites produced during MA fermentation (Fig 6). The concentrations of amino acids rapidly increased in all MA samples, regardless of fermentation temperature, during the early fermentation period. Some amino acids, such as alanine, aspartate, glutamate, and glycine, showed steady increases until the end of fermentation, while other amino acids, such as arginine, leucine, glutamine, isoleucine, threonine, and serine, gradually decreased after reaching their maximum levels, especially at higher fermentation temperatures, at the end of the fermentation period. In particular, arginine significantly decreased at higher fermentation temperatures at the end of fermentation. The overall concentrations of amino acids, including glutamic acid and aspartate that are well known as important components of umami taste in fish sauces, were a little higher in the MA samples fermented at higher temperatures than at lower temperatures.

**Fig 7:**
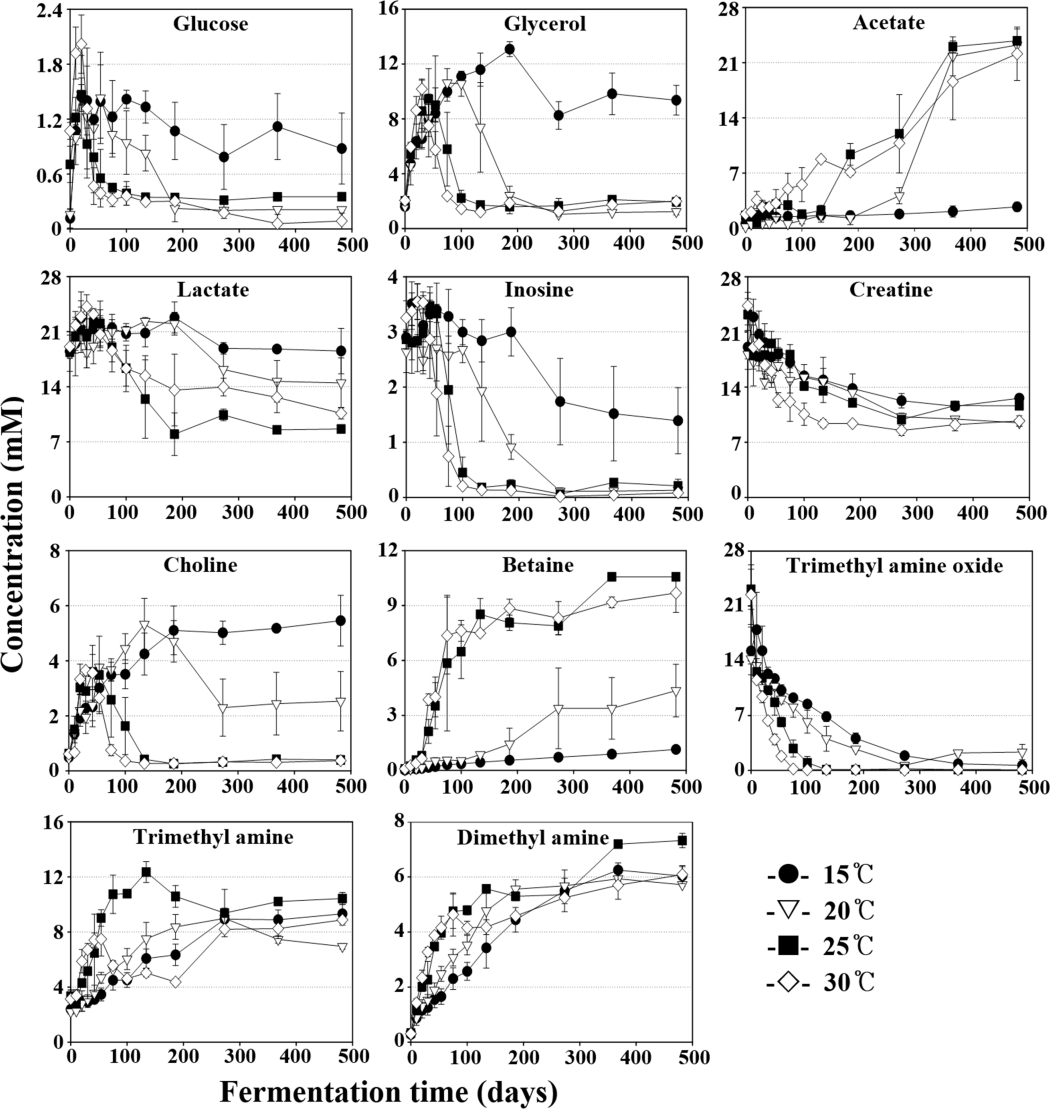
Changes of major organic compounds in MA samples fermented at 15°C, 20°C, 25°C, and 30°C during the entire fermentation period. Data are given as the average value ± standard deviation, measured in triplicate. Quantification was determined using the Chenomx NMR Suite v. 6.1 with 2,2-dimethyl-2-silapentane-5-sulfonate as the internal standard.

The metabolite analysis also showed that glucose, glycerol, acetate, lactate, inosine, creatine, choline, betaine, and methyl amines were major organic compounds in the MA samples (Fig 7). Glucose, probably derived from glycogen in anchovy muscles, increased quickly to around 1–2 mM in all MA samples during the initial fermentation period and then decreased gradually. The glucose decreases occurred more quickly at higher temperatures than lower temperatures, and its level remained almost the same until the end of fermentation in the 15°C MA samples. Glycerol, probably produced by lipid hydrolysis, increased quickly in MA samples at all temperature during an early fermentation period. The glycerol increase was maintained for a longer period at lower temperatures, although its rate was a little lower. The glycerol level increased maximally to approximately 13.4 mM in the 15°C MA samples at 186 days, while it increased maximally to approximately 10.5 mM in the 30°C MA samples of 30 days. After reaching their maximum levels, the glycerol levels decreased. Acetate, which might have been derived from the fermentation of carbohydrates (glucose) or lipids (glycerol), increased gradually as fermentation progressed. The acetate increases occurred more quickly at higher temperatures, but their final concentrations were almost the same in MA samples at all temperature, except for the 15°C MA samples. The acetate in the 15°C MA samples increased only slightly over the entire fermentation period. The faster decreases in glycerol at higher temperatures might be due to the faster increases in acetate, suggesting that acetate may be produced by glycerol fermentation. Lactate, probably derived from anchovy muscles, was identified as a major organic acid in the MA samples, and interestingly, the lactate levels decreased gradually throughout the fermentation period after their initial short increases. The lactate decreases occurred more slowly at lower temperatures.

Inosine, an ATP-hydrolyzed compound produced by enzymes in fish or bacteria [32], decreased in MA samples at all temperature throughout the fermentation period after the initial short increases, and the decreases occurred more rapidly at higher temperatures. Creatine, a nitrogenous organic acid that lends a bitter taste to fish sauce [33], was detected as a major organic compound, and it decreased gradually over the entire fermentation period. Choline increased quickly in all MA samples during the early fermentation period and then began to decrease after reaching their maximum levels. The choline decreases occurred for a longer period in the lower temperature MA samples, similar to what was observed for glycerol. Betaine, a major compatible solute (osmoprotectant) that is produced by choline oxidation in halophilic bacteria [34, 35], increased rapidly during an early fermentation period. The betaine increases occurred more quickly in the higher temperature MA samples, and they occurred only slightly over the entire fermentation in the 15°C MA samples. The faster and greater decreases in choline at higher temperatures might be due to the faster and greater production of betaine by choline oxidation at higher temperatures.

Methylamines including trimethylamine (TMA) and dimethylamine (DMA), which cause the unique odors of fermented fish sauces, are produced by the reduction and/or demethylation of trimethylamine *N*-oxide (TMAO) [36, 37]. TMAO gradually decreased with an increase in TMA and DMA in all MA samples over the entire fermentation period, and the decreases occurred more quickly at higher temperatures. Biogenic amines, mainly produced by the microbial decarboxylation of amino acids or other nitrogen compounds, are important indicators of the quality of fermented seafood and fish sauces [7, 38, 39]. However, biogenic amines, including putrescine, histamine, tyramine, and cadaverine, were not detected in all MA samples during the fermentation period.

## Discussion

Although fermentation temperature is a key factor that influences fish sauce quality, myeolchi-aekjeot (MA), an anchovy fish sauce, has traditionally been produced in Korea by outdoor fermentations without temperature control [9], which is a main reason for MA products with inconsistent tastes and flavors. Therefore, in this study, the effects of temperature on MA fermentation were investigated by analyzing the bacterial communities and metabolites present in MA samples fermented at different temperatures (15°C, 20°C, 25°C, and 30°C).

The qPCR analysis showed that bacterial abundances during MA fermentation differed depending on fermentation temperature (Fig 1B). More bacterial growth occurred at higher temperatures, which suggests that bacteria may contribute more to MA fermentation at higher temperatures. Although the bacterial abundance in MA samples fermented at 15°C was lower than that in the initial MA samples (day 0), Fig 1B clearly shows that bacterial growth occurred at 15°C after an initial decrease in bacterial abundance, suggesting that bacterial population can contribute to MA fermentation even at 15°C.

Bacterial community analysis revealed that the initial bacterial community and overall bacterial successional patterns were a little different from results from a previous analysis of MA fermentation [7] (Fig 5B), which might be because of raw anchovies with different bacterial flora. These results may suggest that different initial bacterial populations in raw anchovies are also one of the main causes of inconsistent quality among MA products, and the use of starter cultures will be required to produce standardized MA products with uniform quality. A previous study reported that in fish fermentation using shrimp (saeu-jeot fermentation), changes in the bacterial community and metabolites occurred similarly, regardless of the fermentation temperature, although shrimp fermentation progressed at different rates depending on temperatures [9]. However, in our anchovy (myeolchi-aekjeot) fermentation, bacterial community and metabolite changes occurred differently depending on fermentation temperatures (Figs 3 and 4), suggesting that the effects of temperature on fish fermentation are different depending on fish. The production of amino acids from proteins during seafood fermentation is important because amino acids are related to fish sauce tastes (umami) and flavors [40]. In shrimp fermentation, the concentration of amino acids at 15°C was higher than those at 10°C, 20°C, or 25°C, indicating that saeu-jeot fermentation may be more appropriate at relatively low fermentation temperature (approximately 15°C) [9]. However, our metabolite analysis in anchovy fermentation showed that amino acids were the major metabolites that appeared during fermentation and that the concentrations of amino acids produced were higher at high temperatures (Fig 6). These results suggest that MA fermentation at higher temperature (25°C and 30°C) may be more desirable, in the view of amino acids produced.

During the initial fermentation period (days 0–10), the genera *Photobacterium* and *Mycoplasma*, including potentially pathogenic members, were identified as dominant in all MA samples [41, 42]. At high temperatures (25°C and 30°C), *Photobacterium* and *Mycoplasma* were not detected after 30 days of fermentation, while at low temperatures (15°C and 20°C), they, especially *Mycoplasma*, which contains many notorious pathogens, were detected through the end of fermentation (Fig 5B), which suggests that low temperature (15°C and 20°C) MA fermentation may not be appropriate in terms of the safety of MA products. However, in shrimp fermentation, the initially dominant genera including *Vibrio*, *Photobacterium*, *Aliivibrio*, and *Enterovibrio*, which may contain potentially pathogenic strains, almost disappeared after 105 days even at 15°C [9], which suggests that the effects of temperature on bacterial successions are quite different depending on fish.

The genus *Chromohalobacter*, including halophilic, gram-negative, motile and rod-shaped bacteria [43], was identified as predominant during the middle of MA fermentation in MA samples at all temperature (Fig 5B). Some members of *Chromohalobacter* have been reported to produce organic acids such as acetate and butyrate from diverse carbohydrates including glucose and glycerol via the Embden-Meyerhof and Entner-Doudoroff pathways [39, 44]. The decrease of organic carbons, including glucose, glycerol, and inosine, and the production of acetate generally correlated well with the abundance of *Chromohalobacter* (Figs 5B and 7), which suggests that *Chromohalobacter* may play an important role, such as acetate production from glycerol, during MA fermentation. In addition, members of the genus *Chromohalobacter*, including *Chromohalobacter beijerinckii*, have the capacity to produce biogenic amines by the decarboxylation of amino acids or other nitrogen compounds, especially in low pH conditions [39, 45]. However, in our study, biogenic amines were not detected in any of the MA samples over the entire fermentation period, which suggests that either *Chromohalobacter* species in the MA samples did not have decarboxylation ability to produce biogenic amines or the biogenic amine production was suppressed by the relatively high pH levels of the MA samples, especially at high temperatures and with abundant *Chromohalobacter* (Fig 1). Although biogenic amines were not detected during MA fermentation in this study, fermentation of MA at 25°C may provide more opportunities for the growth of other *Chromohalobacter* species that can produce biogenic amines.

*Tetragenococcus*, a genus of halophilic lactic acid bacteria that can produce lactate from glucose [11, 46], was identified as a major population during MA fermentation (Fig 5B). Some *Tetragenococcus* species are reported to have the ability to produce biogenic amines during fish sauce fermentation [47–49]. However, because many *Tetragenococcus* species have suppressive effects in biogenic amine production during fish sauce fermentation, members of *Tetragenococcus* as starter cultures have been applied to produce high-quality fish-sauce products [50, 51]. However, lactate concentrations decreased rapidly, despite the abundance of *Tetragenococcus* at higher temperatures, which suggests that *Tetragenococcus* may not be responsible for the production of lactate in MA fermentation and that studies on the roles of *Tetragenococcus* species during MA fermentation are necessary. Because *Tetragenococcus* was predominant in the 30°C MA samples and was not detected in the 15°C MA samples, 30°C fermentation with a greater abundance of *Tetragenococcus* may be more ideal. However, a previous study showed that high temperatures (20°C and 25°C) in shrimp fermentation may not be appropriate due to the growth of *Halanaerobium*, likely responsible for the production of acetate, butyrate, and methylamines [9].

*Alkalibacillus* and *Lentibacillus* were detected at the end of the MA fermentation process (Fig 5). *Alkalibacillus* was identified as dominant at high temperatures (25°C and 30°C), while *Lentibacillus* was detected as dominant at low temperatures, which may be a result of differences in their optimum growth temperatures. The growth of *Alkalibacillus* and *Lentibacillus* and the decrease of some amino acids correlated well (Figs 5B and 6), suggesting that *Alkalibacillus* and *Lentibacillus* may be responsible for the metabolism of certain amino acids [52, 53], and that there may be an optimal MA fermentation time to produce high quality MA products.

In conclusion, we suggest that MA fermentation at 15°C and 20°C may not be appropriate, because of the presence of *Photobacterium* and *Mycoplasma* including some potentially pathogenic species, and the low productions of amino acids. *Tetragenococcus* was more prevalent at 30°C, while *Chromohalobacter* was more prevalent at 25°C. These results suggest that higher temperatures (probably 30°C) might be more appropriate for the production of safe and tasty MA containing lactic acid bacteria, which is contrasted with a previous report that fermentation at relatively low temperatures (around 15°C) might be more suitable for the production of safe and tasty saeu-jeot (fermented salted-shrimp) [9] and this suggests that the effects of temperature on fermentation of salted fish are different depending on fish. This is the first study to reveal the effects of temperature on microbial community and metabolite changes in MA fermentation, and our results may suggest an appropriate fermentation temperature to produce high quality MA. However, because MA fermentation can be different depending on anchovy size, fishing time and place, and storing conditions prior to fermentation, further researches on the microbial communities, metabolites, and sensory characteristics (taste, flavor, and food safety) in MA fermentation using diverse anchovies will be indispensable to the production of safe, high quality MA.

## Supporting Information

S1 File **Contains Fig A and Tables A and B. Fig A:** Principal-component analysis (PCA) of MA samples incubated at 15°C, 20°C, 25°C, and 30°C using 16S rRNA gene sequence data sets with the removal of singletons. **Table A:** List of adapter and barcode sequences in PCR primer sets used in this study. **Table B:** Summary and statistical bacterial diversities of pyrosequencing data derived from myeolchi-aekjeot samples incubated at 15°C, 20°C, 25°C, and 30°C.
(DOCX)
