# Supplementary material for: Effects of Temperature on Bacterial Communities and Metabolites during Fermentation of Myeolchi-Aekjeot, a Traditional Korean Fermented Anchovy Sauce
Source: PLoS One. 2016 Mar 15;11(3):e0151351. doi: 10.1371/journal.pone.0151351 (PMC4792383; doi:10.1371/journal.pone.0151351)
Supplement: S1 File — Contains Fig A and Tables A and B. Fig A: Principal-component analysis (PCA) of MA samples incubated at 15°C, 20°C, 25°C, and 30°C using 16S rRNA gene sequence data sets with the removal of singletons. Table A: List of adapter and barcode sequences in PCR primer sets used in this study. Table B: Summary and statistical bacterial diversities of pyrosequencing data derived from myeolchi-aekjeot samples incubated at 15°C, 20°C, 25°C, and 30°C. (DOCX) [file pone.0151351.s001.docx]

**Supporting information S1 File**

**Fig A.** **Principal-component analysis (PCA) of MA samples incubated at 15°C, 20°C, 25°C, and 30°C using 16S rRNA gene sequence data sets with the removal of singletons.** Numbers beside the symbols represent the fermentation time (days) of MA samples. The curved arrows indicate the routes of the bacterial community changes during the fermentation period in MA samples fermented at different temperatures.


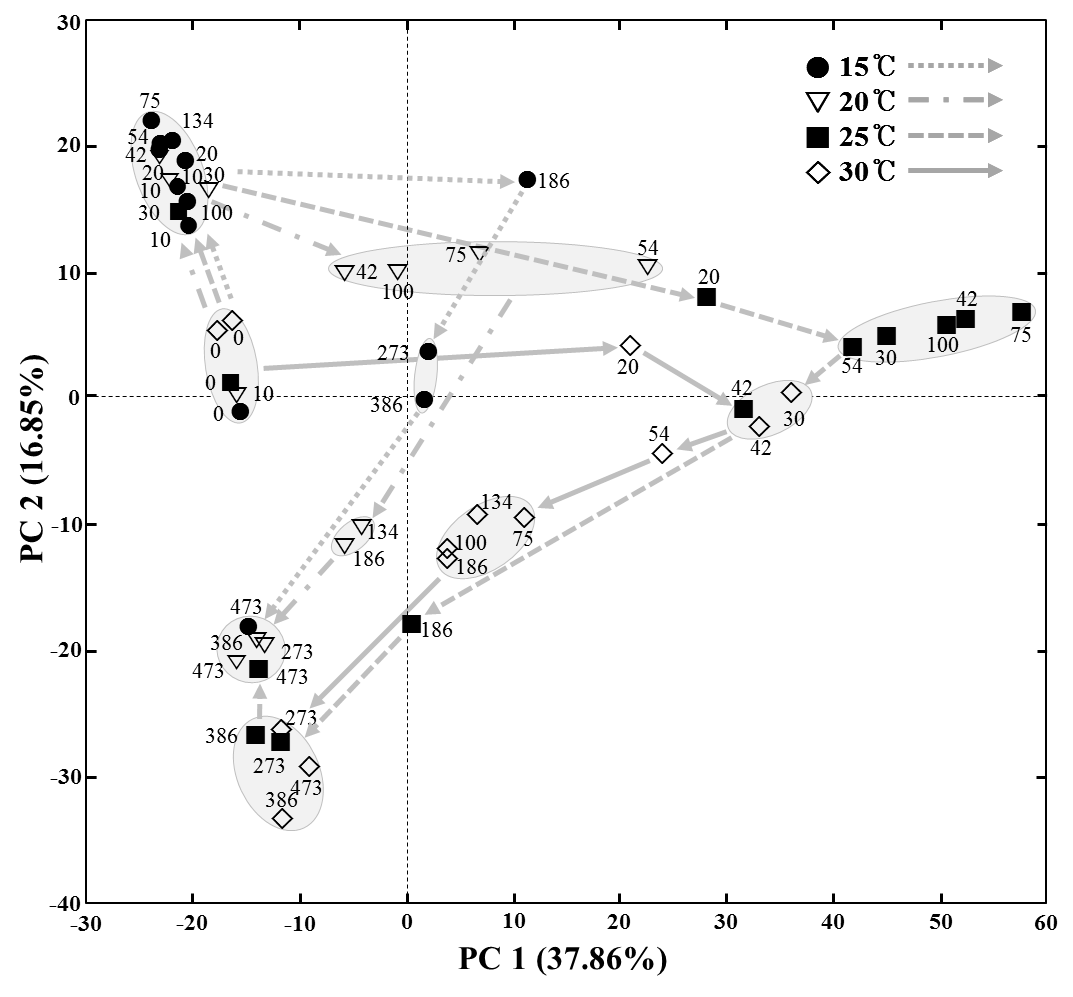


**Table A.** **List of adapter and barcode sequences in PCR primer sets used in this study.**

| Subject | | Sequence (5'-3') | Reference |
| --- | --- | --- | --- |
| Temp. | Days |  |  |
| 15°C | 0 | TCGTCAT | This study |
|  | 10 | AGAGCTG |  |
|  | 20 | ACTGAGT |  |
|  | 30 | TCAGATG |  |
|  | 42 | CGATGAG |  |
|  | 54 | CTGTGAT |  |
|  | 75 | TCTGCAG |  |
|  | 100 | AGCGATG |  |
|  | 134 | ATGCTGAG |  |
|  | 186 | TACAGCAG |  |
|  | 273 | TCGTCAT |  |
|  | 386 | AGAGCTG |  |
|  | 473 | ACTGAGT |  |
| 20°C | 0 | ATCGTGTG | This study |
|  | 10 | CTACACAG |  |
|  | 20 | TAGCTACG |  |
|  | 30 | TCGAGTAG |  |
|  | 42 | TGACTCTG |  |
|  | 54 | ACACTGTG |  |
|  | 75 | ACTACACGC |  |
|  | 100 | ACTCGTATC |  |
|  | 134 | CGTGTACTG |  |
|  | 186 | CTGTCTACG |  |
|  | 273 | TACAGCAG |  |
|  | 386 | ATCGTGTG |  |
|  | 473 | CTACACAG |  |
| 25°C | 0 | AGTCACTAG | This study |
|  | 10 | AGCTCACTG |  |
|  | 20 | ATACGTACG |  |
|  | 30 | CACTATGTG |  |
|  | 42 | CACACGATAG |  |
|  | 54 | ATGTGTCTAG |  |
|  | 75 | ATGTACGATG |  |
|  | 100 | ATCGTCTGTG |  |
|  | 134 | ATCGTAGCAG |  |
|  | 186 | ATCACGTGCG |  |
|  | 273 | ACTACACGC |  |
|  | 386 | CGTGTACTG |  |
|  | 473 | ATACGTACG |  |
| 30°C | 0 | AGTCTGACTG | This study |
|  | 10 | AGCTATCGCG |  |
|  | 20 | TCATATACGCG |  |
|  | 30 | TAGATAGTGCG |  |
|  | 42 | ACGTCTCTACG |  |
|  | 54 | CTAGAGACACT |  |
|  | 75 | TCGCTAGTGTA |  |
|  | 100 | TACGCTCTCGT |  |
|  | 134 | CTCGATAGACG |  |
|  | 186 | AGACAGTACAG |  |
|  | 273 | ATGTGTCTAG |  |
|  | 386 | ATCGTCTGTG |  |
|  | 473 | AGTCTGACTG |  |
| Adapter sequence | |  |  |
| A adapter | | CCATCTCATCCCTGCGTGTCTCCGACTCAG | Roesch et al.  (2007) |
| B adapter | | CCTATCCCCTGTGTGCCTTGGCAGTCTCAG |  |

**Table B. Summary and statistical bacterial diversities of pyrosequencing data derived from myeolchi-aekjeot samples incubated at 15°C, 20°C, 25°C, and 30°C.**

| Sample | Day | Total reads | Clean reads | OTU* | Shannon* | Chao1* | Evenness* |
| --- | --- | --- | --- | --- | --- | --- | --- |
| 15°C | 0 | 269 | 152 | 27 | 2.73 | 38.0 | 0.828 |
|  | 10 | 2802 | 1557 | 182 | 3.15 | 447.0 | 0.606 |
|  | 20 | 2626 | 1362 | 190 | 3.21 | 424.0 | 0.611 |
|  | 30 | 3229 | 1578 | 214 | 3.52 | 436.1 | 0.656 |
|  | 42 | 3747 | 2038 | 203 | 3.04 | 421.0 | 0.572 |
|  | 54 | 2336 | 1368 | 164 | 2.99 | 326.9 | 0.586 |
|  | 75 | 2829 | 1571 | 158 | 2.74 | 332.8 | 0.541 |
|  | 100 | 2927 | 1545 | 378 | 4.07 | 1018.0 | 0.685 |
|  | 134 | 2703 | 1545 | 174 | 2.94 | 388.2 | 0.570 |
|  | 186 | 2181 | 1250 | 128 | 2.58 | 278.2 | 0.533 |
|  | 273 | 247 | 213 | 39 | 2.77 | 56.0 | 0.756 |
|  | 386 | 7320 | 4049 | 79 | 0.71 | 208.4 | 0.162 |
|  | 473 | 6065 | 5354 | 66 | 1.91 | 74.3 | 0.455 |
| 20°C | 0 | 1887 | 935 | 105 | 3.27 | 232.2 | 0.703 |
|  | 10 | 2861 | 1653 | 193 | 3.10 | 415.3 | 0.589 |
|  | 20 | 2142 | 1097 | 174 | 3.11 | 377.5 | 0.602 |
|  | 30 | 3070 | 1311 | 179 | 3.24 | 377.8 | 0.625 |
|  | 42 | 3129 | 1289 | 168 | 3.22 | 396.4 | 0.628 |
|  | 54 | 2611 | 1199 | 110 | 2.45 | 222.0 | 0.522 |
|  | 75 | 2709 | 1152 | 120 | 2.72 | 245.1 | 0.568 |
|  | 100 | 2684 | 1004 | 97 | 2.57 | 172.3 | 0.561 |
|  | 134 | 1809 | 1218 | 51 | 1.90 | 133.7 | 0.484 |
|  | 186 | 2661 | 1680 | 52 | 2.37 | 59.3 | 0.600 |
|  | 273 | 5654 | 4946 | 146 | 2.80 | 189.0 | 0.562 |
|  | 386 | 2002 | 1789 | 83 | 2.68 | 118.4 | 0.606 |
|  | 473 | 10855 | 9589 | 154 | 2.58 | 186.5 | 0.511 |
| 25°C | 0 | 1020 | 500 | 41 | 2.48 | 79 | 0.668 |
|  | 10 | 1352 | 287 | 64 | 2.82 | 193 | 0.678 |
|  | 20 | 2582 | 1500 | 80 | 1.98 | 143.1 | 0.452 |
|  | 30 | 2004 | 1193 | 20 | 1.04 | 56.0 | 0.348 |
|  | 42 | 2187 | 1242 | 29 | 0.96 | 47.2 | 0.284 |
|  | 54 | 2272 | 1449 | 24 | 1.47 | 29.0 | 0.46 |
|  | 75 | 2832 | 1803 | 27 | 0.73 | 104.9 | 0.222 |
|  | 100 | 2451 | 1264 | 34 | 0.97 | 47.0 | 0.276 |
|  | 134 | 2334 | 1599 | 61 | 2.04 | 78.8 | 0.497 |
|  | 186 | 2216 | 1461 | 78 | 2.17 | 103.6 | 0.498 |
|  | 273 | 7323 | 2960 | 152 | 2.93 | 218.5 | 0.584 |
|  | 386 | 3943 | 3382 | 135 | 2.85 | 182.3 | 0.582 |
|  | 473 | 2276 | 2004 | 111 | 2.83 | 174.1 | 0.601 |
| 30°C | 0 | 4445 | 1995 | 245 | 3.61 | 501.1 | 0.656 |
|  | 10 | 5195 | 1882 | 360 | 4.34 | 774.6 | 0.738 |
|  | 20 | 2626 | 1177 | 143 | 2.80 | 318.6 | 0.563 |
|  | 30 | 1521 | 954 | 29 | 1.76 | 35.0 | 0.524 |
|  | 42 | 2321 | 1496 | 52 | 1.72 | 66.6 | 0.435 |
|  | 54 | 1709 | 1243 | 45 | 2.02 | 64.0 | 0.532 |
|  | 75 | 1766 | 1177 | 46 | 2.04 | 54.8 | 0.534 |
|  | 100 | 2206 | 1624 | 54 | 1.98 | 75.0 | 0.496 |
|  | 134 | 2834 | 1891 | 62 | 2.38 | 70.6 | 0.577 |
|  | 186 | 1176 | 753 | 43 | 2.53 | 73.0 | 0.671 |
|  | 273 | 2932 | 2037 | 83 | 2.67 | 134.7 | 0.600 |
|  | 386 | 9415 | 1982 | 73 | 2.03 | 92.7 | 0.473 |
|  | 473 | 8380 | 7196 | 162 | 2.42 | 236.4 | 0.476 |

Abbreviation: OTU, operational taxonomic unit.

*Diversity indices of the bacterial communities were calculated using the RDP pyrosequencing pipeline based on the 16S rRNA gene sequencing reads.

**Reference**

1. Roesch LF, Fulthorpe RR, Riva A, Casella G, Hadwin AK, et al. (2007) Pyrosequencing enumerates and contrasts soil microbial diversity. ISME J 1: 283-290. doi:10.1038/ismej.2007. Pubmed: 53 18043639.
